# (3,4-Dihy­droxy­oxolan-2-yl)methyl 4-methyl­benzene­sulfonate

**DOI:** 10.1107/S1600536810044788

**Published:** 2010-11-06

**Authors:** Sergey Dibrov, Maia Carnevali, Thomas Hermann

**Affiliations:** a Department of Chemistry and Biochemistry, University of California, San Diego, 9500 Gilman Drive, La Jolla, CA 92093-0358, USA

## Abstract

The racemic title compound, C_12_H_16_O_6_S, possesses a five-membered ring that adopts an envelope-shaped conformation; the two hy­droxy groups occupy quasi-axial positions. Adjacent mol­ecules are linked by O—H⋯O hydrogen bonds to generate a ribbon that runs along the *a* axis of the ortho­rhom­bic unit cell. The crystal studied was an inversion twin.

## Related literature

For the synthesis of the title compound, see: Kapitan & Grazca (2008 ▶); Park *et al.* (2005 ▶). For the use of xylitol tosyl­ates in the synthesis of bicyclic oxetanes, see: Köll & Oetling (1987 ▶).
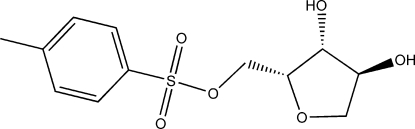


## Experimental

### 

#### Crystal data

C_12_H_16_O_6_S
*M* *_r_* = 288.31
Orthorhombic, 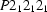

*a* = 5.414 (4) Å
*b* = 10.172 (8) Å
*c* = 24.080 (18) Å
*V* = 1326.0 (17) Å^3^
*Z* = 4
Mo *K*α radiation
μ = 0.26 mm^−1^
*T* = 173 K
0.50 × 0.30 × 0.20 mm

#### Data collection

Bruker APEXII CCD diffractometer
Absorption correction: multi-scan (*SADABS*; Bruker, 2005 ▶) *T*_min_ = 0.879, *T*_max_ = 0.949
14329 measured reflections
3154 independent reflections
2333 reflections with *I* > 2σ(*I*)
*R*_int_ = 0.056

#### Refinement

*R*[*F* ^2^ > 2σ(*F* ^2^)] = 0.047
*wR*(*F* ^2^) = 0.110
*S* = 1.02
3154 reflections
177 parameters
H-atom parameters constrained
Δρ_max_ = 0.26 e Å^−3^
Δρ_min_ = −0.19 e Å^−3^
Absolute structure: Flack (1983 ▶), 1174 Friedel pairs
Flack parameter: 0.47 (12)

### 

Data collection: *APEX2* (Bruker, 2005 ▶); cell refinement: *SAINT* (Bruker, 2005 ▶); data reduction: *SAINT*; program(s) used to solve structure: *SHELXS97* (Sheldrick, 2008 ▶); program(s) used to refine structure: *SHELXL97* (Sheldrick, 2008 ▶); molecular graphics: *SHELXTL* (Sheldrick, 2008 ▶); software used to prepare material for publication: *SHELXTL*.

## Supplementary Material

Crystal structure: contains datablocks I, global. DOI: 10.1107/S1600536810044788/ng5055sup1.cif

Structure factors: contains datablocks I. DOI: 10.1107/S1600536810044788/ng5055Isup2.hkl

Additional supplementary materials:  crystallographic information; 3D view; checkCIF report

## Figures and Tables

**Table 1:** Hydrogen-bond geometry (Å, °)

*D*—H⋯*A*	*D*—H	H⋯*A*	*D*⋯*A*	*D*—H⋯*A*
O2—H2⋯O1^i^	0.84	2.03	2.834 (4)	160
O3—H3⋯O3^ii^	0.84	2.17	2.931 (2)	151

Symmetry codes: (i) 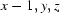; (ii) 
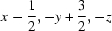
.

**Fig. 1.**
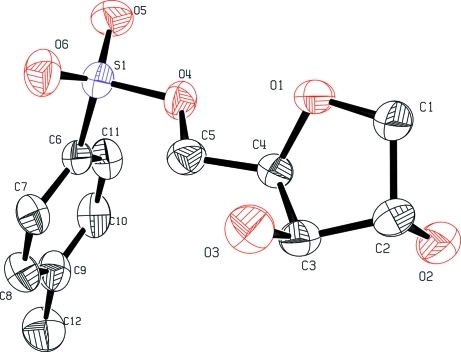
The molecular structure of title compound, with atom labels and 50% probability displacement ellipsoids for non-H atoms.

**Fig. 2.**
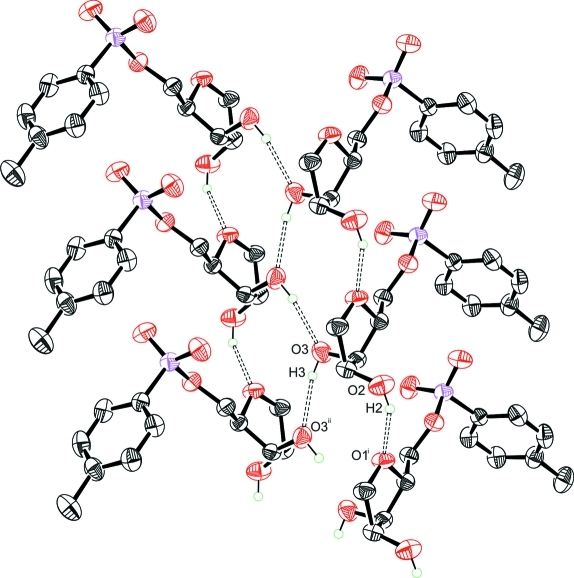
Hydrogen bonding showing the formation of dimeric chains. Intermolecular hydrogen bonds are shown as dashed lines. Symmetry codes: (i) x - 1, y, z; (ii) x - 1/2, -y + 3/2, -z.

**Fig. 3.**
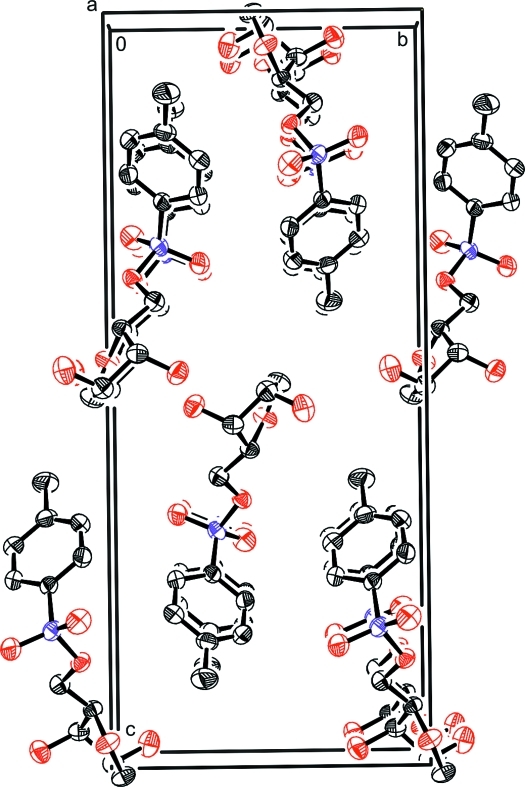
The crystal packing of the title compound, viewed down the a axis.

## supplementary crystallographic information

### Comment

The title compound is the product of the cyclization of xylitol in the presence of *p*-toluenesulfonyl chloride. The synthetic procedure is described below. We report here the single-crystal X-ray structure.

Asymmetric unit of the title compound is composed of molecule 1 (Fig. 1). Molecules are linked together into infinite chains by the intermolecular O2—H2···O1^i^ (Fig. 2) that form dimers by the O3—H3···O3^ii^ hydrogen bonds. These dimeric chains propagate along the *a* axis (Fig. 3).

### Experimental

In our attempt to make 2,3,4-trihydroxypentane-1,5-diyl bis(4-methylbenzenesulfonate) we have obtained (3,4-dihydroxytetrahydrofuran-2-yl)methyl 4-methylbenzenesulfonate as a major product. The title compound has been synthesized by modification of procedures described by Park *et al.* (2005) and Kapitan & Grazca (2008). To a solution of racemic Xylitol (33 mmol) in pyridine (40 ml), *p*-toluenesulfonylchloride (69 mmol) in pyridine was added drop wise at -10°C. The reaction mixture was kept at 4°C overnight, resulting in formation of white precipitate which was removed by filtration. The filtrate was poured over 300 ml of water and kept in an ice bath for 20 minutes. Extraction with dichloromethane, followed by washing of the organic layer with saturated NaCl solution, drying over sodium sulfate, filtration and evaporation yielded the title compound which was finally purified by flash chromatography. In a sample vial, 20 mg of compound was taken and dissolved in MeOH. Upon slow evaporation at 273 K the crystals are formed as colorless blocks.

### Refinement

H atoms were positioned geometrically and refined using a riding model with C—H = 0.95–1.00 Å and O—H = 0.84 Å with *U*_iso_(H) = 1.2*U*_eq_. The Flack parameter refined to nearly 0.5, in agreement with the racemic nature of the Xylitol reactant.

### Crystal data

**Table:**

C_12_H_16_O_6_S	*F*(000) = 608
*M**_r_* = 288.31	*D*_x_ = 1.444 Mg m^−^^3^
Orthorhombic, *P*2_1_2_1_2_1_	Mo *K*α radiation, λ = 0.71073 Å
Hall symbol: P2ac2ab	Cell parameters from 3862 reflections
*a* = 5.414 (4) Å	θ = 2.6–23.4°
*b* = 10.172 (8) Å	µ = 0.26 mm^−^^1^
*c* = 24.080 (18) Å	*T* = 173 K
*V* = 1326.0 (17) Å^3^	Block, colourless
*Z* = 4	0.50 × 0.30 × 0.20 mm

### Data collection

**Table:**

Bruker APEXII CCD diffractometer	3154 independent reflections
Radiation source: fine-focus sealed tube	2333 reflections with *I* > 2σ(*I*)
graphite	*R*_int_ = 0.056
φ and ω scans	θ_max_ = 28.5°, θ_min_ = 1.7°
Absorption correction: multi-scan (*SADABS*; Bruker, 2005)	*h* = −7→7
*T*_min_ = 0.879, *T*_max_ = 0.949	*k* = −13→13
14329 measured reflections	*l* = −31→26

### Refinement

**Table:**

Refinement on *F*^2^	Hydrogen site location: inferred from neighbouring sites
Least-squares matrix: full	H-atom parameters constrained
*R*[*F*^2^ > 2σ(*F*^2^)] = 0.047	*w* = 1/[σ^2^(*F*_o_^2^) + (0.0413*P*)^2^ + 0.5008*P*] where *P* = (*F*_o_^2^ + 2*F*_c_^2^)/3
*wR*(*F*^2^) = 0.110	(Δ/σ)_max_ = 0.003
*S* = 1.01	Δρ_max_ = 0.26 e Å^−^^3^
3154 reflections	Δρ_min_ = −0.19 e Å^−^^3^
177 parameters	Extinction correction: *SHELXL97* (Sheldrick, 2008), Fc^*^=kFc[1+0.001xFc^2^λ^3^/sin(2θ)]^-1/4^
0 restraints	Extinction coefficient: 0.0054 (12)
Primary atom site location: structure-invariant direct methods	Absolute structure: Flack (1983), with 1174 Friedel pairs
Secondary atom site location: difference Fourier map	Flack parameter: 0.47 (12)

### Special details

**Table:**

Geometry. All e.s.d.'s (except the e.s.d. in the dihedral angle between two l.s. planes) are estimated using the full covariance matrix. The cell e.s.d.'s are taken into account individually in the estimation of e.s.d.'s in distances, angles and torsion angles; correlations between e.s.d.'s in cell parameters are only used when they are defined by crystal symmetry. An approximate (isotropic) treatment of cell e.s.d.'s is used for estimating e.s.d.'s involving l.s. planes.
Refinement. Refinement of *F*^2^ against ALL reflections. The weighted *R*-factor *wR* and goodness of fit *S* are based on *F*^2^, conventional *R*-factors *R* are based on *F*, with *F* set to zero for negative *F*^2^. The threshold expression of *F*^2^ > σ(*F*^2^) is used only for calculating *R*-factors(gt) *etc*. and is not relevant to the choice of reflections for refinement. *R*-factors based on *F*^2^ are statistically about twice as large as those based on *F*, and *R*- factors based on ALL data will be even larger.

### Fractional atomic coordinates and isotropic or equivalent isotropic displacement parameters (Å^2^)

**Table:**

	*x*	*y*	*z*	*U*_iso_*/*U*_eq_	
S1	0.49846 (15)	0.83391 (7)	0.19055 (3)	0.03938 (19)	
O4	0.3443 (4)	0.9149 (2)	0.14690 (8)	0.0403 (5)	
O3	−0.1096 (4)	0.7717 (2)	0.02146 (9)	0.0522 (6)	
H3	−0.2351	0.7308	0.0104	0.063*	
O5	0.6954 (4)	0.9196 (2)	0.20441 (10)	0.0558 (6)	
O1	0.1981 (4)	0.9922 (2)	0.03798 (8)	0.0452 (5)	
O6	0.5516 (4)	0.7081 (2)	0.16814 (8)	0.0533 (6)	
C3	−0.1840 (5)	0.8929 (3)	0.04656 (12)	0.0393 (7)	
H3A	−0.3360	0.8827	0.0698	0.047*	
C5	0.1566 (5)	0.8449 (3)	0.11540 (12)	0.0397 (6)	
H5A	0.0364	0.8031	0.1409	0.048*	
H5B	0.2338	0.7755	0.0924	0.048*	
O2	−0.3312 (4)	1.1107 (2)	0.02581 (11)	0.0574 (6)	
H2	−0.4753	1.0904	0.0355	0.069*	
C9	−0.0409 (6)	0.8029 (3)	0.33240 (11)	0.0477 (8)	
C11	0.2748 (6)	0.9224 (3)	0.28326 (13)	0.0469 (8)	
H11	0.3752	0.9984	0.2793	0.056*	
C4	0.0298 (5)	0.9430 (3)	0.07931 (11)	0.0354 (6)	
H4	−0.0271	1.0181	0.1029	0.042*	
C10	0.1045 (7)	0.9131 (3)	0.32584 (12)	0.0518 (9)	
H10	0.0872	0.9842	0.3511	0.062*	
C7	0.1562 (6)	0.7062 (3)	0.25310 (12)	0.0431 (7)	
H7	0.1760	0.6342	0.2284	0.052*	
C2	−0.2121 (6)	0.9991 (3)	0.00288 (13)	0.0429 (7)	
H2A	−0.2984	0.9661	−0.0311	0.051*	
C6	0.2955 (5)	0.8185 (3)	0.24657 (11)	0.0359 (6)	
C12	−0.2297 (8)	0.7949 (4)	0.37844 (14)	0.0703 (11)	
H12A	−0.3449	0.7228	0.3708	0.105*	
H12B	−0.3211	0.8779	0.3806	0.105*	
H12C	−0.1457	0.7788	0.4138	0.105*	
C1	0.0535 (5)	1.0362 (3)	−0.00883 (12)	0.0458 (8)	
H1A	0.1114	0.9932	−0.0433	0.055*	
H1B	0.0689	1.1325	−0.0134	0.055*	
C8	−0.0126 (7)	0.6997 (3)	0.29599 (11)	0.0478 (7)	
H8	−0.1106	0.6229	0.3004	0.057*	

### Atomic displacement parameters (Å^2^)

**Table:**

	*U*^11^	*U*^22^	*U*^33^	*U*^12^	*U*^13^	*U*^23^
S1	0.0386 (3)	0.0384 (4)	0.0411 (3)	0.0040 (4)	−0.0029 (4)	0.0060 (3)
O4	0.0423 (11)	0.0401 (11)	0.0385 (10)	0.0032 (10)	−0.0059 (9)	0.0067 (9)
O3	0.0547 (13)	0.0456 (13)	0.0562 (13)	−0.0053 (11)	−0.0067 (12)	−0.0107 (11)
O5	0.0423 (12)	0.0619 (15)	0.0633 (14)	−0.0097 (12)	−0.0080 (11)	0.0074 (12)
O1	0.0308 (11)	0.0596 (14)	0.0452 (12)	−0.0038 (10)	−0.0002 (9)	0.0146 (10)
O6	0.0641 (15)	0.0438 (12)	0.0518 (12)	0.0142 (11)	0.0056 (11)	0.0022 (10)
C3	0.0351 (15)	0.0448 (18)	0.0381 (14)	−0.0006 (14)	0.0023 (13)	0.0003 (13)
C5	0.0376 (15)	0.0412 (16)	0.0404 (14)	−0.0025 (14)	−0.0027 (13)	−0.0007 (14)
O2	0.0416 (12)	0.0519 (14)	0.0788 (16)	0.0049 (11)	0.0027 (13)	0.0074 (12)
C9	0.059 (2)	0.0514 (19)	0.0331 (13)	0.0089 (17)	0.0039 (14)	0.0064 (14)
C11	0.061 (2)	0.0351 (17)	0.0441 (16)	0.0009 (15)	−0.0106 (15)	0.0002 (14)
C4	0.0322 (15)	0.0393 (15)	0.0347 (13)	−0.0035 (13)	0.0008 (12)	0.0030 (11)
C10	0.075 (2)	0.0412 (18)	0.0396 (16)	0.0087 (17)	−0.0040 (16)	−0.0052 (15)
C7	0.0569 (19)	0.0326 (15)	0.0397 (15)	−0.0016 (15)	0.0018 (15)	0.0015 (13)
C2	0.0352 (16)	0.0484 (19)	0.0452 (16)	−0.0016 (14)	−0.0071 (14)	0.0028 (15)
C6	0.0396 (15)	0.0337 (15)	0.0345 (13)	0.0022 (13)	−0.0036 (12)	0.0031 (12)
C12	0.084 (3)	0.076 (3)	0.0504 (19)	0.009 (2)	0.023 (2)	0.001 (2)
C1	0.0361 (18)	0.061 (2)	0.0401 (15)	0.0023 (14)	−0.0006 (13)	0.0114 (15)
C8	0.0600 (18)	0.0426 (16)	0.0407 (14)	−0.0031 (18)	0.0032 (16)	0.0053 (12)

### Geometric parameters (Å, °)

**Table:**

S1—O5	1.417 (2)	C9—C10	1.379 (5)
S1—O6	1.418 (2)	C9—C12	1.510 (4)
S1—O4	1.575 (2)	C11—C10	1.382 (5)
S1—C6	1.747 (3)	C11—C6	1.382 (4)
O4—C5	1.454 (3)	C11—H11	0.9500
O3—C3	1.430 (4)	C4—H4	1.0000
O3—H3	0.8400	C10—H10	0.9500
O1—C4	1.439 (3)	C7—C6	1.378 (4)
O1—C1	1.443 (3)	C7—C8	1.381 (4)
C3—C4	1.491 (4)	C7—H7	0.9500
C3—C2	1.516 (4)	C2—C1	1.513 (4)
C3—H3A	1.0000	C2—H2A	1.0000
C5—C4	1.491 (4)	C12—H12A	0.9800
C5—H5A	0.9900	C12—H12B	0.9800
C5—H5B	0.9900	C12—H12C	0.9800
O2—C2	1.418 (4)	C1—H1A	0.9900
O2—H2	0.8400	C1—H1B	0.9900
C9—C8	1.376 (4)	C8—H8	0.9500
			
O5—S1—O6	119.47 (15)	C5—C4—H4	108.8
O5—S1—O4	103.55 (13)	C3—C4—H4	108.8
O6—S1—O4	109.01 (12)	C9—C10—C11	121.4 (3)
O5—S1—C6	110.27 (14)	C9—C10—H10	119.3
O6—S1—C6	109.91 (14)	C11—C10—H10	119.3
O4—S1—C6	103.24 (13)	C6—C7—C8	119.2 (3)
C5—O4—S1	117.54 (17)	C6—C7—H7	120.4
C3—O3—H3	109.5	C8—C7—H7	120.4
C4—O1—C1	107.7 (2)	O2—C2—C1	107.8 (3)
O3—C3—C4	107.4 (2)	O2—C2—C3	110.3 (3)
O3—C3—C2	110.4 (2)	C1—C2—C3	102.2 (2)
C4—C3—C2	101.6 (2)	O2—C2—H2A	112.0
O3—C3—H3A	112.3	C1—C2—H2A	112.0
C4—C3—H3A	112.3	C3—C2—H2A	112.0
C2—C3—H3A	112.3	C7—C6—C11	121.1 (3)
O4—C5—C4	107.3 (2)	C7—C6—S1	120.5 (2)
O4—C5—H5A	110.2	C11—C6—S1	118.4 (2)
C4—C5—H5A	110.2	C9—C12—H12A	109.5
O4—C5—H5B	110.2	C9—C12—H12B	109.5
C4—C5—H5B	110.2	H12A—C12—H12B	109.5
H5A—C5—H5B	108.5	C9—C12—H12C	109.5
C2—O2—H2	109.5	H12A—C12—H12C	109.5
C8—C9—C10	118.9 (3)	H12B—C12—H12C	109.5
C8—C9—C12	120.1 (3)	O1—C1—C2	107.0 (2)
C10—C9—C12	121.0 (3)	O1—C1—H1A	110.3
C10—C11—C6	118.4 (3)	C2—C1—H1A	110.3
C10—C11—H11	120.8	O1—C1—H1B	110.3
C6—C11—H11	120.8	C2—C1—H1B	110.3
O1—C4—C5	110.1 (2)	H1A—C1—H1B	108.6
O1—C4—C3	104.2 (2)	C9—C8—C7	120.9 (3)
C5—C4—C3	115.9 (2)	C9—C8—H8	119.5
O1—C4—H4	108.8	C7—C8—H8	119.5
			
O5—S1—O4—C5	167.5 (2)	C4—C3—C2—C1	36.7 (3)
O6—S1—O4—C5	39.3 (2)	C8—C7—C6—C11	2.2 (4)
C6—S1—O4—C5	−77.5 (2)	C8—C7—C6—S1	−176.4 (2)
S1—O4—C5—C4	177.38 (18)	C10—C11—C6—C7	−2.1 (4)
C1—O1—C4—C5	154.6 (2)	C10—C11—C6—S1	176.5 (2)
C1—O1—C4—C3	29.6 (3)	O5—S1—C6—C7	−152.8 (2)
O4—C5—C4—O1	66.7 (3)	O6—S1—C6—C7	−19.1 (3)
O4—C5—C4—C3	−175.4 (2)	O4—S1—C6—C7	97.1 (3)
O3—C3—C4—O1	74.7 (3)	O5—S1—C6—C11	28.5 (3)
C2—C3—C4—O1	−41.3 (3)	O6—S1—C6—C11	162.3 (2)
O3—C3—C4—C5	−46.5 (3)	O4—S1—C6—C11	−81.6 (2)
C2—C3—C4—C5	−162.5 (2)	C4—O1—C1—C2	−5.7 (3)
C8—C9—C10—C11	0.8 (5)	O2—C2—C1—O1	96.4 (3)
C12—C9—C10—C11	−178.9 (3)	C3—C2—C1—O1	−19.8 (3)
C6—C11—C10—C9	0.6 (5)	C10—C9—C8—C7	−0.7 (5)
O3—C3—C2—O2	168.6 (2)	C12—C9—C8—C7	178.9 (3)
C4—C3—C2—O2	−77.6 (3)	C6—C7—C8—C9	−0.7 (5)
O3—C3—C2—C1	−77.0 (3)		

### Hydrogen-bond geometry (Å, °)

**Table:**

*D*—H···*A*	*D*—H	H···*A*	*D*···*A*	*D*—H···*A*
O2—H2···O1^i^	0.84	2.03	2.834 (4)	160
O3—H3···O3^ii^	0.84	2.17	2.931 (2)	151

Symmetry codes: (i) *x*−1, *y*, *z*; (ii) *x*−1/2, −*y*+3/2, −*z*.
